# Das EKG beim Leistungssportler und Athleten: Was ist normal und was nicht?

**DOI:** 10.1007/s00399-022-00917-0

**Published:** 2023-01-20

**Authors:** Amaar Ujeyl, David Niederseer

**Affiliations:** 1 Praxis LANS Cardio, Hamburg, Deutschland; 2 MSc Sports Cardiology, St. George’s University of London, London, Großbritannien; 3 Klinik für Kardiologie, Universitäres Herzzentrum Zürich, Universitätsspital Zürich, Universität Zürich, Rämistrasse 100, Zürich, 8091 Schweiz

**Keywords:** Elektrokardiogramm, Sportlerherz, Plötzlicher Herztod, Sportkardiologie, Sport, Electrocardiogram, Athlete’s heart, Sudden cardiac death, Sports cardiology, Elite sports

## Abstract

**Hintergrund:**

Das Elektrokardiogramm (EKG) hat sich als mobiles und kostengünstiges Verfahren zur präventiven Risikostratifizierung von Amateur- und Leistungssportlern im Rahmen der Sporttauglichkeitsuntersuchung etabliert. Zentrales Ziel ist dabei die Senkung der Fälle des plötzlichen Herztods im Sport durch eine Früherkennung der häufigsten zugrundeliegenden kardialen Erkrankungen wie hereditärer Kardiomyopathien, primärer Arrhythmien, aber auch der koronaren Herzerkrankung bei Master-Athleten.

**Methoden:**

Durch kontinuierliche Weiterentwicklung der erstmals 2010 von der Europäischen Gesellschaft für Kardiologie (ESC) vorgestellten EKG-Kriterien konnte die Trennschärfe zur Unterscheidung physiologischer, trainingsbedingter kardialer Adaptationen des Sportlerherzens, die im EKG erkennbar werden, und relevanten kardialen Pathologien stetig verbessert werden. Auf diese Weise ließ sich das Risiko von falsch-positiven Befunden und fälschlicher Stigmatisierung von Athleten unterschiedlichen Alters und unterschiedlicher Ethnizität stetig senken.

**Schlussfolgerung:**

Der vorliegende Artikel zeichnet den Wandel der EKG-Kriterien im Lichte der wachsenden wissenschaftlichen Evidenz der vergangenen ca. 15 Jahre nach, stellt die zentralen Botschaften der aktuell geltenden „internationalen“ EKG-Kriterien aus dem Jahr 2017 vor und erarbeitet, welche Herausforderungen bei der EKG-Befundung von Amateur- und Leistungssportlern weiterhin Gegenstand der Forschung sind.

## Abstract (en)

**Background:**

The electrocardiogram (ECG) has become a mobile and cost-effective routine assessment tool to risk stratify leisure-time and professional athletes during preparticipation screening. A central goal is the reduction of sudden cardiac death in sports through early recognition of the most prevalent underlying cardiac pathologies, e.g., hereditary cardiomyopathies or primary arrhythmias.

**Methods:**

Continuous evolution of the first ECG criteria for athletes, presented in 2010 by the European Society of Cardiology (ESC), has helped to improve the specificity of the criteria to both detect cardiac pathologies in early stages and differentiate from physiologic adaptation of the athlete’s heart. Thus, the risk of false-positive findings and erroneous stigmatizations of athletes has been successfully reduced.

**Conclusion:**

This review article intends to trace back the changes of the ECG criteria in the light of a growing body of scientific evidence over the last 15 years, to present the key messages of the current International ECG criteria from 2017 and to identify some of the remaining challenges that wait to be answered by physicians in the field of sports medicine and sports cardiology.

Die häufigste nichttraumatische Todesursache von jungen Athleten (< 35 Jahre) während des Sports ist mit 75 % aller Todesfälle der plötzliche Herztod. Kardiomyopathien und primär elektrische Erkrankungen zählen zu den häufigsten zugrundeliegenden Erkrankungen für ein erhöhtes Risiko des plötzlichen Herztods bei jungen Sportlern. Dazu zählen hypertrophe Kardiomyopathien (HCM), Formen der arrhythmogenen (rechts)ventrikulären Kardiomyopathie (AVC), Ionenkanalerkrankungen (z. B. Brugada-Syndrom, Long- bzw. Short-QT-Syndrom) sowie akzessorische Leitungsbahnen (z. B. Wolf-Parkinson-White[WPW]-Syndrom; [1–3]; Abb. 1).

**Figure 1:**
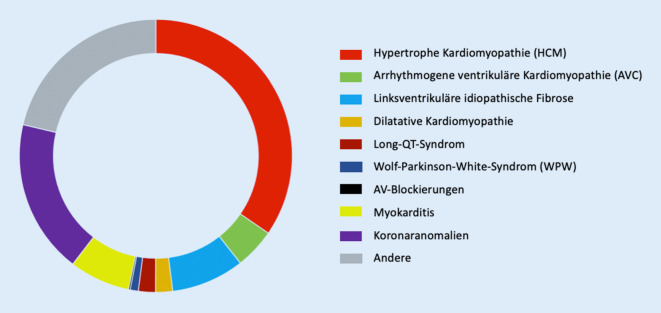

Die Mehrheit dieser Erkrankungen bleibt bei Athleten bis zum todbringenden Ereignis klinisch stumm und entgeht daher einer allein durch Symptome getriggerten Diagnostik [4]. Präventiv angelegte Screeningkonzepte in Sportvereinen und im Profisport dienen der Früherkennung von Athleten mit erhöhtem Risiko. Als mobiles, kostengünstiges und flächendeckend verfügbares Screeningtool bietet sich das EKG in diesem Kontext an. Das Ruhe-EKG galt jedoch lange als wenig aussagekräftig für das Screening von Athleten aufgrund einer fraglichen Spezifität der Befunde. Die Hauptsorge konzentrierte sich dabei auf die mögliche hohe Zahl an falsch-positiven Befunden, mit dem Risiko von hohen Kosten durch vermeidbare Folgeuntersuchungen, psychologischen Traumatisierungen der Athleten und unbegründeten Disqualifikationen. Genährt wurde dieses Misstrauen gegenüber dem Ruhe-EKG durch den Umstand, dass die Beurteilung der EKGs häufig durch Sportmediziner und allgemeine Kardiologen ohne Spezialkenntnisse erfolgte, auf der Basis individueller Erfahrungshorizonte und unter Bezug auf traditionelle EKG-Kriterien für die Normalbevölkerung. Lange mangelte es an konkreten Leitlinien zur korrekten Interpretation des EKGs bei Athleten. Erfahrungen aus der Region Veneto in Italien zeigten erstmals systematisch das Potenzial eines EKG-zentrierten Screeningmodells bei Athleten auf. Über einen Zeitraum von 25 Jahren und durch Screenings von > 33.000 Athleten konnte die Inzidenz des plötzlichen Herztods bei jungen Athleten in der Region um 90 % (von 3,6 auf 0,4/100.000) reduziert werden ([5]; Abb. 2).

**Figure 2:**
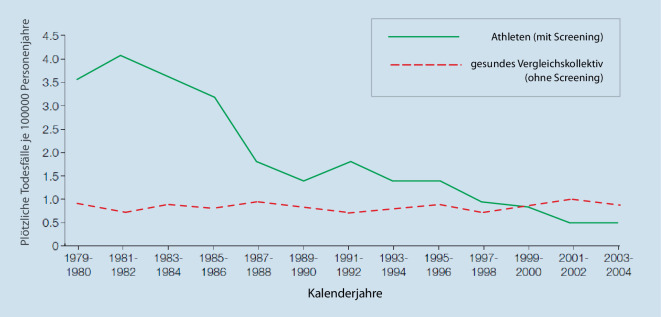

Diese Ergebnisse unterstützten die Hypothese, dass insbesondere asymptomatische Frühformen von hereditären Kardiomyopathien und Arrhythmien in der Regel bereits initial mit EKG-Veränderungen einhergehen (im Sinne einer Genotyp-Phänotyp-Kaskade; [6, 7]). So weist die Mehrheit der an spezifischen Kardiomyopathien Erkrankten begleitende EKG-Auffälligkeiten auf (95 % der Individuen mit HCM und 80 % der Betroffenen mit AVC; [8, 9]). Das EKG gilt zudem als Goldstandard zur primären diagnostischen Abklärung von arrhythmogenen Erkrankungen wie dem WPW-Syndrom, Ionenkanalerkrankungen wie dem Brugada-Syndrom oder dem Long- und Short-QT-Syndrom. Zu den als pathologisch bewerteten EKG-Veränderungen gehören Störungen der Repolarisation wie T‑Negativierungen, ST-Streckensenkungen, pathologische Q‑Zacken, Erregungsleitungsstörungen wie dem kompletten Linksschenkelblock, Zeichen der ventrikulären Präexzitation (Delta-Welle), verlängerte bzw. verkürzte QT-Intervalle oder Brudaga-typische Repolarisationsstörungen [10].

Trotz der nachgewiesenen Effektivität als Screeningtool stellt die Interpretation von EKG-Befunden bei Athleten eine Herausforderung dar. EKG-Veränderungen sind bei dieser Patientengruppe insgesamt häufig und meist Ausdruck eines physiologischen elektrischen und strukturellen Umbaus des Herzens („cardiac remodeling“) bzw. Folge der Anpassungen des autonomen Nervensystems durch regelmäßiges sportliches Training (definiert als Trainingsvolumen > 4 h/Woche; [11, 12]). Schätzungsweise bis zu 60 % aller gesunden Athleten weisen isoliert oder in Kombination entsprechende EKG-Veränderungen auf. Dazu zählen Sinusbradykardien, Sinusarrhythmien, AV-Blockierungen I°, Zeichen der frühen Repolarisation („early repolarisation“), Rechtsschenkelblockbilder und EKG-Zeichen der linksventrikulären Hypertrophie (LVH). Die Prävalenz und das Ausmaß dieser Veränderungen ist abhängig von der Herkunft und dem Geschlecht der Athleten, der Sportart sowie der Trainingsintensität und dem Trainingsvolumen. Ziel eines EKG-zentrierten Screeningverfahrens ist es daher, mithilfe eines validierten Kriterienkatalogs diejenigen EKG-Veränderungen, die auf potenziell tödliche kardiovaskuläre Erkrankungen hinweisen, von harmlosen physiologischen Veränderungen durch regelmäßiges sportliches Training möglichst sicher zu unterscheiden.

## Evolution der EKG-Kriterien im wissenschaftlichen Diskurs

Eine Fülle von wissenschaftlichen Publikationen, vor allem Kohortenstudien von Athleten, hat im vergangenen Jahrzehnt unser Verständnis für die korrekte Interpretation von spezifischen EKG-Befunden in unterschiedlichsten Athletenkollektiven (mit Unterschieden in Geschlecht, Ethnizität, Alter der Athleten, Sportart und -intensität) erweitert. Die Kriterien wurden daraufhin in den vergangenen Jahren sukzessive angepasst, um die Trennschärfe zur Unterscheidung zwischen pathologischen Prozessen einerseits und physiologischen Anpassungen des Herzens andererseits zu verbessern.

### ESC-Kriterien von 2005 und 2010

Im Jahr 2005 veröffentlichte die Europäische Gesellschaft für Kardiologie (ESC) erstmals ein Konsensuspapier zum kardiovaskulären Screening junger Athleten. Kernstück der Empfehlungen war die Auflistung von EKG-Kriterien, die als potenziell pathologisch angesehen wurden und bei deren Nachweis bei Athleten eine weitergehende Abklärung sinnvoll erschien [13]. Anschließende Validierungsstudien zeigten jedoch die Unzulänglichkeiten dieser ersten Kriterien, da die Anwendung dazu führte, dass bis zu 50 % der EKGs von Athleten als abklärungswürdig klassifiziert wurden. Zur Verbesserung der Spezifität und Kosteneffektivität wurden die Kriterien 2010 von der ESC weitergehend präzisiert [14]. EKG-Veränderungen wurden dabei unterteilt in solche, die als üblich und trainingsbedingt eingeschätzt wurden, und solche, die als unüblich und nicht trainingsbedingt kategorisiert wurden und eine klärende Diagnostik erforderten.

**Figure 3:**
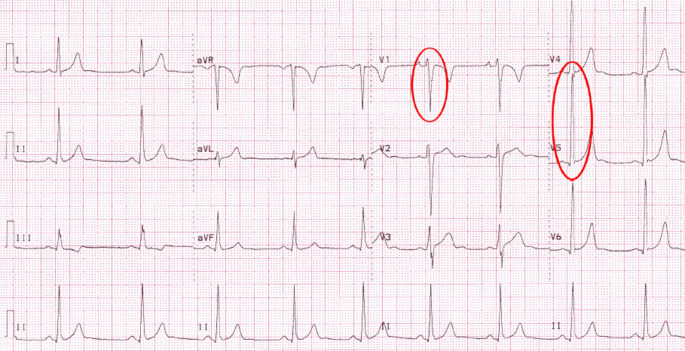

**Figure 4:**
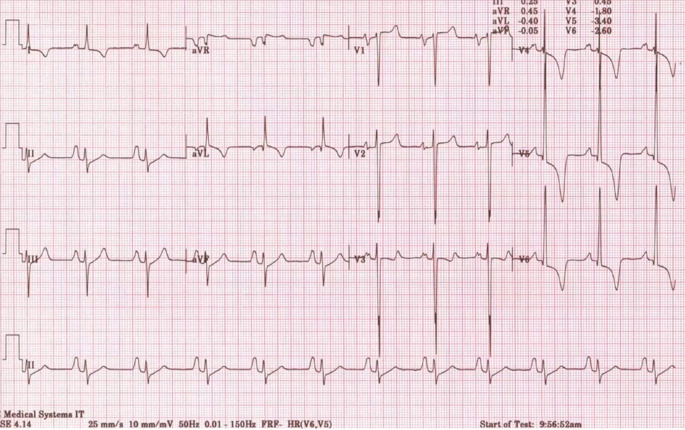

Diese Neubewertung betraf beispielsweise auch das isoliert auftretende EKG-Zeichen einer LVH (Abb. 3). Während dieses nach den ESC-Kriterien von 2005 zum Ausschluss einer möglichen zugrundeliegenden HCM noch als abklärungswürdig galt, zeigten darauffolgende Studien, dass isolierte Zeichen der LVH im EKG bei bis zu 45 % der gesunden Athleten und 10 % der Athletinnen auftraten. Im Gegensatz dazu wiesen lediglich 1,8 % der an HCM Erkrankten isolierte EKG-Zeichen einer LVH auf, stattdessen waren sie in diesem Kontext häufig zusammen mit weiteren Auffälligkeiten im EKG (wie Zeichen der rechts-, links- bzw. biatrialen Dilatation, Herzachsenabweichung mit überdrehtem Linkstyp und Repolarisationsstörungen im Sinne von T‑Negativierungen bzw. ST-Streckensenkungen) zu finden (Abb. 4; [11]).

Konsequenterweise wurden isolierte Zeichen der LVH in den ESC-Kriterien von 2010 nicht mehr in der Liste der abklärungswürdigen EKG-Befunde aufgeführt. In der Summe führten die Änderungen der ESC-Kriterien von 2010 bereits zu einer substanziellen Reduzierung falsch-positiver Befunde, deren Häufigkeit mit bis zu 10 % jedoch weiterhin unbefriedigend hoch war.

Als Schwäche der ESC-Kriterien von 2005 und 2010 stellte sich heraus, dass sie in der Mehrheit auf Daten einer im Wesentlichen ethnisch homogenen Gruppe europäischstämmiger („Weißer“; [15]) und männlicher Athleten basierte und somit der ethnischen und geschlechtlichen Diversität der sportlichen Gemeinschaft nicht ausreichend Rechnung trug [16]. Dies führte in der Vergangenheit zu einer inakzeptabel hohen Rate an falsch-positiven Befunden unter Athleten afroamerikanisch/afrokaribischer Herkunft (in der Folge als „Schwarze Athleten“ bezeichnet; die Großschreibung von „Schwarz“ und „Weiß“ orientiert sich an der angloamerikanischen Original-Literatur und verdeutlicht die politische Dimension dieser Begrifflichkeiten [15, 17]), meist getriggert durch nachweisbare T‑Negativierungen [18]. Aufgrund einer ethnisch bedingten genetischen Disposition zeigen diese Athleten ein ausgeprägteres elektrisches Remodelling im Rahmen des regelmäßigen athletischen Trainings. Bis zu zwei Drittel der Schwarzen Athleten weisen Repolarisationsstörungen im Ruhe-EKG auf und bis zu 25 % T‑Negativierungen. Die Prävalenz von T‑Negativierungen in den anterioren Ableitungen (V1–V4) erwies sich in einer Kohortenstudie von Papadakis et al. mit 12,7 % aller gesunden Schwarzen Athleten ohne Nachweis einer kardialen Pathologie als ungewöhnlich hoch gegenüber lediglich 4 % bei Weißen Athleten [18].

### Seattle-Kriterien (2013) und „Refined“-Kriterien (2014)

**Figure 5:**
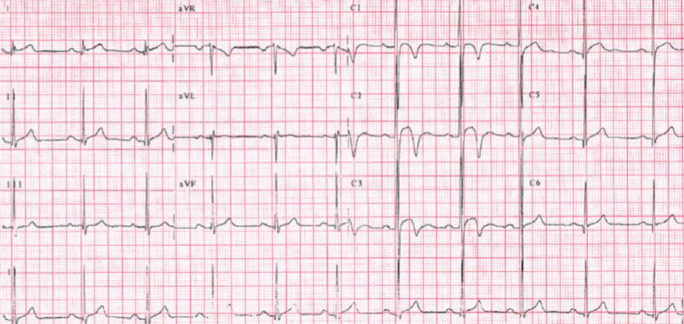

Diese Erkenntnisse wurden in den 2013 publizierten *Seattle-Kriterien* berücksichtigt, die erstmals auf der Expertise eines transatlantischen Expertengremiums beruhte [19]. Mit der Einführung von ethnisch spezifischen EKG-Kriterien erfolgte eine Neubewertung der T‑Negativierung in den anterioren Ableitungen (V1–V4), die ab sofort bei Schwarzen Athleten als Normvariante galten (Abb. 5). Durch die Anwendung der Seattle-Kriterien konnte in verschiedenen Kollektiven von Athleten eine deutliche Senkung der falsch-positiven Befunde bei gleichzeitiger Bewahrung der Spezifität für die Erkennung von kardiovaskulären Erkrankungen nachgewiesen werden. Bereits 2014 erfolgte eine weitere wichtige Anpassung der EKG-Kriterien („refined criteria“), welche die Ergebnisse zweier Kohortenstudien zur Prävalenz von EKG-Veränderungen bei gesunden Athleten berücksichtigte [20, 21]. Bisher als pathologisch bewertete EKG-Kriterien wie Zeichen der rechts- und linksatrialen Dilatation, Herzachsenabweichungen (überdrehter Links- bzw. Rechtstyp) und Zeichen der rechtsventrikulären Hypertrophie wurden einer neuen Befundkategorie zugeordnet und als „grenzwertig“ (borderline) entschärft [22]. Diese Borderline-Kriterien wurden fortan nur dann als abklärungswürdig bzw. pathologisch angesehen, wenn mindestens zwei von ihnen nachzuweisen waren. Dies führte zu einer weiteren Reduktion von *pathologischen* EKGs in einer Validierungsstudie von über 5000 Athleten (11,5 % bei Schwarzen und 5,3 % bei Weißen Athleten). Im direkten Vergleich mit den ursprünglichen ESC-Kriterien aus 2005 erhöhten diese „Refined“-Kriterien die Spezifität bei Schwarzen Athleten von 40,3 auf 82,4 % und bei Weißen Athleten von 73,8 auf 94,1 % ohne Einbußen bei der Erkennung von relevanten kardiovaskulären Erkrankungen [22].

## Status quo – die „internationalen“ EKG-Kriterien (2017)

**Figure 6:**
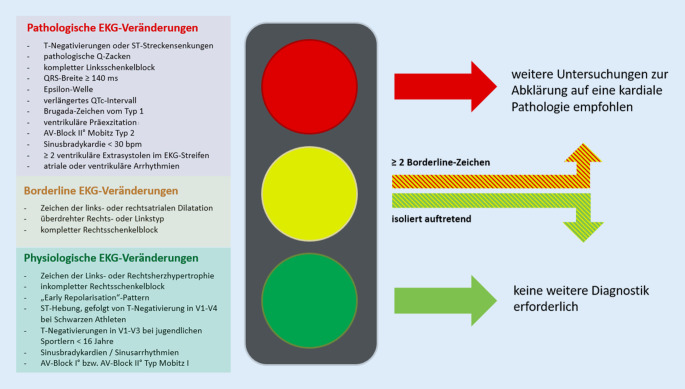

Die aktuell geltenden „internationalen Empfehlungen zur EKG-Interpretation bei Athleten“ stammen aus dem Jahr 2017 [10]. Neu eingeführt wurde u. a. ein anschauliches Ampelsystem der wichtigsten EKG-Befunde in *Grün* (physiologisch), *Gelb* (borderline) und *Rot* (pathologisch) (Abb. 6), die Einführung eines jugendlichen Musters („juvenile pattern“) für Athleten unter 16 Jahren und eine Neubewertung von T‑Negativierungen in den anterioren Ableitungen V1 *und* V2. Bislang galten EKGs von Weißen Athleten mit T‑Negativierungen jenseits der Ableitung V1 bereits als abklärungswürdig. Auf der Grundlage einer großen Studie von Malhotra et al. mit > 14.000 Athleten, die eine Prävalenz von anterioren T‑Negativierungen von 1,8 % bei gesunden Weißen Athleten ohne kardiale Pathologien nachwies, wurde diese Empfehlung angepasst [23].

### Aktuelle Kriterien

**Figure 7:**
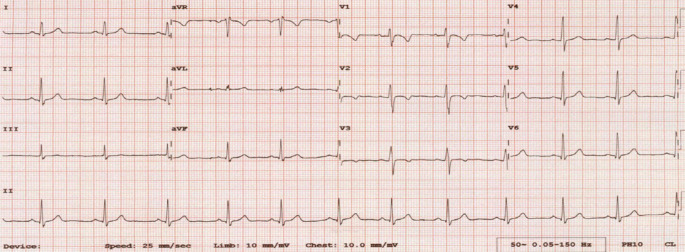

Im Folgenden wird eine Übersicht über die wichtigsten aktuellen Kriterien gegeben:

- Isolierte Zeichen der rechts- und linksventrikulären Hypertrophie gelten als physiologische Anpassung an die trainingsbedingte Vergrößerung der Herzhöhlen sowie der myokardialen Masse.
- Ein inkompletter Rechtsschenkelblock ist Folge der physiologischen kardialen Adaptation und erfordert keine weitere Abklärung.
- T‑Negativierungen in den anterioren Ableitungen V1–V3 stellen bei beschwerdefreien Athleten < 16 Jahren eine Normvariante dar („juvenile pattern“; Abb. 7).
- T‑Negativierungen in den Ableitungen V1–V4, insbesondere im Zusammenhang mit einer J‑Punkt-Erhöhung und konvex verlaufenden ST-Streckenhebungen sind bei Schwarzen Athleten als Normvariante der Repolarisation anzusehen.
- Herzachsenabweichungen (überdrehter Links- bzw. Rechtstyp), Zeichen der atrialen Dilatation (rechts-, links- oder biatrial) und ein kompletter Rechtsschenkelblock sind, soweit sie isoliert auftreten, als physiologisch anzusehen und gelten nur im Verbund (zwei oder mehr Zeichen) als abklärungsbedürftig.
- ST-Streckensenkungen > 0,05 mV in zwei oder mehr Ableitungen gelten als pathologisch und bedürfen einer weiteren Abklärung.
- Die Definition einer pathologischen Q‑Zacke wurde angepasst und orientiert sich zukünftig am Verhältnis von Q‑ und R‑Zacke (≥ 1/4 des Q/R-Verhältnisses oder QR-Breite ≥ 40 ms).
- Ein kompletter Linksschenkelblock erfordert immer eine umfassende diagnostische Abklärung inklusive Echokardiographie und Magnetresonanztomographie (MRT) des Herzens mit Beurteilung der Myokardperfusion,
- T‑Negativierungen in den lateralen Ableitungen (I, aVL, V5–V6) sollten unabhängig von der Herkunft des Athleten immer umfassend abgeklärt werden zum Ausschluss einer zugrundeliegenden Kardiomyopathie
- Bei Athleten mit tiefen T‑Negativierungen (> 0,2 mV) und ST-Streckensenkungen in den lateralen oder inferolateralen Ableitungen sollte eine Kardio-MRT diagnostischer Standard sein zum Ausschluss einer apikalen Form der hypertrophen Kardiomyopathie, die der Diagnose per Echokardiographie u. U. entgehen kann.
- T‑Negativierungen in den anterioren Ableitungen, die über die Ableitung V2 hinausgehen und ohne begleitende ST-Streckenhebungen bzw. mit begleitenden ST-Streckensenkungen auftreten, sollten bei Athleten ≥ 16 Jahren eine weitere Diagnostik zum Ausschluss einer AVC veranlassen. Weitere EKG-Zeichen, die beim Auftreten von anterioren T‑Negativierungen auf eine AVC hindeuten, sind: Niedervoltage in den Extremitätenableitungen, ein verzögerter Aufstieg der S‑Zacke, ventrikuläre Extrasystolen und eine Epsilon-Welle.
- Athleten mit Zeichen der Präexzitation sollten auch ohne bisherige Beschwerden eine weitergehende Risikostratifizierung erhalten (per Ergometrie und ggf. elektrophysiologischer Untersuchung) zur Erhebung der Leitungseigenschaften der akzessorischen Leitungsbahn.
- Unspezifische intrakardiale Erregungsleitungsverzögerungen mit einer QRS-Breite von ≥ 140 ms erfordern eine weitergehende Abklärung.
- Eine korrigierte QT-Zeit (QTc-Zeit) von ≥ 470 ms bei Athleten und ≥ 480 ms bei Athletinnen sollte auch bei fehlenden Beschwerden und unauffälliger Familienanamnese eine weitergehende Abklärung veranlassen.
- Mehr als eine ventrikuläre Extrasystole (VES) im Ruhe-EKG-Streifen gilt als pathologisch. Eine Inzidenz von ≥ 2000 VES/24 h bzw. das gehäufte Auftreten von VES unter Belastung sollte eine umfassende Abklärung inklusive Kardio-MRT mit Late-Gadolinium-Enhancement (LGE) und ggf. elektrophysiologischer Testung beinhalten, da Athleten mit ≥ 2000 VES/24 h in bis zu 30 % der Fälle eine zugrundliegende strukturelle Erkrankung aufwiesen [24].
- Bei beschwerdefreien Athletinnen und Athleten über 35 Jahre sollten folgende Befunde eine weitergehende Ischämiediagnostik veranlassen: T‑Negativierungen, pathologische Q‑Zacken, kompletter Rechts- oder Linksschenkelblock bzw. linksanteriorer Hemiblock, fehlende R‑Progression und Vorhofflimmern.
- Bei einer Reihe von hereditären Kardiomyopathien wie HCM, AVC und dilatativer Kardiomyopathie können EKG-Veränderungen noch vor dem Auftreten der klinischen Herzinsuffizienz nachgewiesen werden. Bei Sportlern mit EKG-Veränderungen, die auf eine Kardiomyopathie hindeuten (wie z. B. T‑Negativierungen in den lateralen Ableitungen), deren initiale Abklärung aber keinen Hinweis auf Kardiomyopathie zeigt, sollten Verlaufsuntersuchungen erfolgen, die über das Ende der sportlichen Karriere hinausgehen.

### Performance der internationalen Kriterien

Die Weiterentwicklung der EKG-Kriterien hat zu einer beeindruckenden Verbesserung der Spezifität bei der Beurteilung von Athleten geführt. Die Rate an Athleten mit pathologischen EKGs wurde durch die Anpassungen der *Internationalen* Kriterien im Vergleich zu den *Seattle-Kriterien* um 57 % reduziert (von 4,3 % auf 1,9 %), validiert an 11.168 jugendlichen Fußballspielern [25]. Die häufigste EKG-Auffälligkeit bei der Anwendung der *Internationalen* Kriterien waren dabei mit 48 % T‑Negativierungen [25, 26]. Gleichzeitig wurde die Rate an falsch-positiven Befunden von 2,8 % (bei Anwendung der Seattle-Kriterien) auf 1,3 % verringert. Davon profitieren nicht allein sportkardiologische Spezialisten, sondern vor allem auch nichtkardiologisch fokussierte Sportmediziner beim Screening in der Fläche sowie auch das Gesundheitssystem als Ganzes durch eine Verringerung anfallender Kosten.

## Offene Fragen und Ausblick

Kein Zweifel besteht darin, dass auch die aktuellen EKG-Kriterien nur ein Abbild des gegenwärtigen Wissensstands sind und sich in der Zukunft auf dem Boden neuerer Erkenntnisse weiter wandeln werden. Zahlreiche offene Fragen warten noch auf eine wissenschaftliche Antwort durch zukünftige interessierte Sportkardiologen und Elektrophysiologen. Exemplarisch seien nur einige von ihnen an dieser Stelle erwähnt.

### T-Negativierungen

T‑Negativierungen bleiben die relevanteste Veränderung im EKG von Athleten. Sie sind weit verbreitet bei Kardiomyopathien wie der HCM oder der AVC, die mit einem erhöhten Risiko des plötzlichen Herztods einhergehen. Neben der Lokalisation im 12-Kanal-EKG ist die Ethnizität der betroffenen Athleten der wichtigste prognostische Faktor. Während die prognostische Bedeutung von T‑Negativierungen in den lateralen Ableitungen (I, aVL, V5/V6) unbestritten ist, bleibt die Bewertung von T‑Negativierungen in den anterioren Ableitungen (V1–V4) eine Herausforderung, insbesondere im Spannungsfeld der berichteten hohen Prävalenz von T‑Negativierungen in den anterioren Ableitungen bei Schwarzen Athleten und der beabsichtigten Früherkennung von Formen der AVC. Die aktuelle Definition der Normvariante in V1–V4 führt bei Schwarzen Athleten zu einer verringerten Sensitivität zur Erkennung der AVC. Unter Menschen mit afroamerikanisch/afrokaribischer Herkunft gilt der gefundene Weg jedoch als gangbarer Kompromiss zur Vermeidung einer unangemessen hohen Rate an falsch-positiven Befunden in einem Kollektiv von Athleten mit bekannt niedriger AVC-Prävalenz.

Auch bei der Einschätzung von T‑Negativierungen in den Ableitungen V1–V3 von jugendlichen Athleten gilt es, sich zu verdeutlichen, dass die Grenze von 16 Jahren eine der Praktikabilität geschuldete Vereinfachung darstellt und die Normalisierung der Repolarisation im Jugendalter nicht schlagartig erfolgt, sondern eher vom biologischen als vom chronologischen Alter abhängt. Der gewählte Grenzwert von 16 Jahren dient sinnvollerweise nicht als klarer Cut-off-Wert, sondern vielmehr als Orientierung.

### Ventrikuläre Extrasystolie

Bei der bisherigen Beurteilung von VES gilt bislang lediglich ein numerischer Grenzwert von mehr als einer VES im Ruhe-EKG-Streifen bzw. ≥ 2000 VES im 24-Langzeit-EKG als abklärungswürdig. Neuere Daten legen jedoch nahe, dass weniger die numerische VES-Last als vielmehr die Morphologie, die Komplexität der Extrasystolie und das Verhältnis zur körperlichen Anstrengung entscheidende Hinweise auf das zugrundeliegende pathophysiologische Substrat geben [27]. Insbesondere die Morphologie der VES spielt dabei eine zentrale Rolle zur Beurteilung der prognostischen Relevanz. VES mit infundibulärem (Linksschenkelblock-Morphologie und inferiore Achse) oder faszikulärem (Rechtsschenkelblock-Morphologie und schmaler QRS-Komplex) Ursprung gelten in der Regel als prognostisch günstig. Bei VES mit Linksschenkelblockbild und superiorer Achse oder mit Rechtsschenkelblockbild mit breitem QRS-Komplex hingegen liegt die Wahrscheinlichkeit einer Assoziation mit einer zugrundeliegenden Herzmuskelerkrankung höher. Ob als Konsequenz aus diesen Daten die Empfehlung abgeleitet werden kann, dass auch einzelne VES im Ruhe-EKG als abklärungswürdig gelten, wenn sie nicht infundibulären oder faszikulären Ursprungs sind, obliegt den Autoren zukünftiger Empfehlungen.

### Ethnizität

Die dichotome Einteilung in „Weiße“ Athleten einerseits und „Schwarze“ Athleten andererseits stellt eine unzulässige Vereinfachung der Welt des Sports dar und muss perspektivisch auch bei der Beurteilung der EKGs bei Athleten überwunden werden. Neben Menschen mit multiethnischem („mixed-race“) Hintergrund, die nicht in dieses Raster passen, zeigen uns die Daten von ausgeprägten regionalen Unterschieden in der Prävalenz von Repolarisationsvarianten unter Athleten mit west-, zentral- oder ostafrikanischer Herkunft, dass unser aktuelles binäres System nur eine Zwischenstation in unserem Verständnis von EKGs bei Athleten sein kann [17].

## Fazit für die Praxis

- Das Elektrokardiogramm (EKG) allein kann nicht alle Athleten mit erhöhtem Risiko eines plötzlichen Herztods identifizieren.
- Dies betrifft allen voran Athleten mit Koronaranomalien oder – später im Leben – mit koronarer Herzerkrankung.
- Insbesondere bei Athleten über 35 Jahren ist die Effektivität des EKG-basierten Screenings deutlich limitiert.
- Bei kritischer Anwendung der EKG-Kriterien unter Berücksichtigung der Stärken und Schwächen haben Sportkardiologen und Sportmediziner aber bereits heute ein kosteneffizientes, transportables und bewährtes Werkzeug an der Hand, um die Inzidenz des plötzlichen Herztods in jungen Jahren im Rahmen des Sports bedeutend zu senken.
