# Erratum zu: Das EKG beim Leistungssportler und Athleten

**DOI:** 10.1007/s00399-023-00929-4

**Published:** 2023-02-14

**Authors:** Amaar Ujeyl, David Niederseer

**Affiliations:** 1 Praxis LANS Cardio, Hamburg, Deutschland; 2 MSc Sports Cardiology, St. George’s University of London, London, Großbritannien; 3 Klinik für Kardiologie, Universitäres Herzzentrum Zürich, Universitätsspital Zürich, Universität Zürich, Rämistrasse 100, Zürich, 8091 Schweiz

**Erratum zu:**

**Herzschr Elektrophys 2022**

10.1007/s00399-022-00917-0

In diesem Artikel wurde Abb. 2 durch eine aktualisierte Version mit korrigierter Legende ersetzt. Zudem wurden die Abbildungsbeschreibungen für Abb. 2 und 7 wie folgt korrigiert:

**Figure 1:**
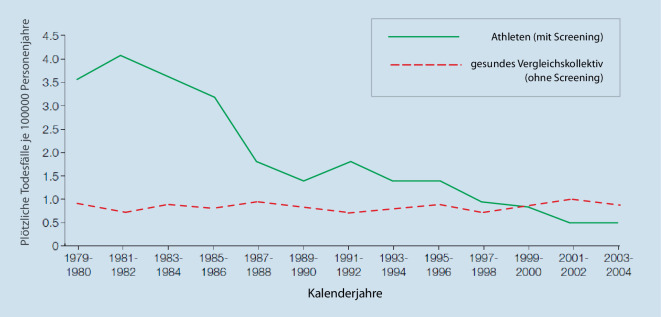

**Figure 2:**
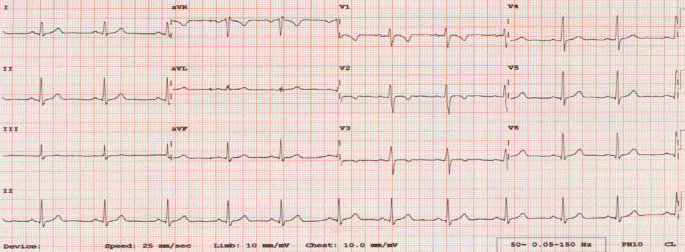

Der Originalbeitrag wurde korrigiert.

